# Efficacy and local irritation evaluation of *Eriobotrya japonica* leaf ethanol extract

**DOI:** 10.1186/s42826-019-0003-3

**Published:** 2019-06-24

**Authors:** Nak-Won Seong, Won-Jun Oh, Il-Soo Kim, Su-Jin Kim, Ji-Eun Seo, Chang-Eon Park, Da-Young Kim, Je-Won Ko, Jong-Choon Kim

**Affiliations:** 1 Health Care Institute, Korea Testing and Research Institute, Hwasun, Jeonnam 58141 Republic of Korea; 2 College of Veterinary Medicine (BK21 Plus Project Team), Chonnam National University, Gwangju, 61186 Republic of Korea

**Keywords:** *Eriobotrya japonica* leaf ethanol extract, Anti-oxidant activity, Anti-inflammatory activity, Anti-melanogenic activity, Local irritation, Animal alternative test

## Abstract

**Background:**

Although *Eriobotrya japonica* leaves have been studied as a raw material for various cosmetic products, little is known about the anti-oxidant, anti-inflammatory, and anti-melanogenic activities of *Eriobotrya japonica* leaf ethanol extract (EJEE).

**Methods:**

This study was conducted to evaluate the anti-oxidant, anti-inflammatory, and anti-melanogenic activities of EJEE using different in vitro models. In addition, we investigated the potential irritation of EJEE to skin and eye using animal alternative tests.

**Results:**

The total content of polyphenols, one of the active constituents of EJEE, was analyzed by high-performance liquid chromatography and found to contain 88.68 mg tannic acid equivalent/g. EJEE showed a concentration-dependent 1,1-diphenyl-2-picrylhydrazyl radical scavenging activity, 2,2′-azino-bis(3-ethylbenzthiazoline-6-sulfonic acid) radical scavenging activity, and a superoxide dismutase-like activity. The anti-inflammatory effect of 0.5% (w/v) EJEE was demonstrated by a reduction in lipopolysaccharide-induced nitric oxide and tumor necrosis factor-alpha levels in RAW 264.7 cells. EJEE also significantly inhibited melanogenesis in melanocyte stimulating hormone-induced B16F1 cells. EJEE did not show any irritation in skin and eye in animal alternative test.

**Conclusions:**

These results indicate that the EJEE possesses anti-oxidant, anti-inflammatory, and anti-melanogenic activities, while it did not induce toxicity or irritation in neither skin nor eye. Therefore, EJEE can be used as a cosmetic ingredient for skin improvement.

## Background

Recently, because of the growing interest in the field of cosmetology, many studies have been conducted with different natural cosmetic agents. There is a growing need for dermatological procedures (skin de-pigmentation, anti-wrinkle treatment, etc.) for various conditions, such as acne and hyperpigmentation [1–3]. Many natural products are increasingly being used as medicinal and cosmetic agents [4]. Free radicals and reactive oxygen species (ROS) are naturally produced in vivo, and their production is augmented by exposure to sunlight and various toxic chemicals. They are considered to be one of the most important target in skin aging research.

Ultraviolet (UV) radiation exposure induces the formation of excess ROS, which can interact with lipids, proteins, and DNA and can alter the cellular functions causing melanogenesis and aging-related disorders [5, 6]. Free radicals and ROS are generated by oxidative stress in skin cells and stimulate melanin synthesis. Previous reports have demonstrated that hyperpigmentation and skin aging are related to the production of nitric oxide (NO) and inflammatory factors [7–9]. NO produced by inducible nitric oxide synthase (iNOS) causes tissue damage in the inflamed areas. iNOS produces a large amount of NO in the macrophages during inflammation in response to a variety of inflammatory stimuli [10]. NO and tumor necrosis factor-α (TNF-α) were reported to play a role not only in inflammation but also in the formation of skin wrinkles and melanin production [8, 9, 11]. Thus, various studies have been actively carried out to evaluate the anti-inflammatory activities of several natural compounds.

The plant named *Eriobotrya japonica* is used in the traditional system of medicine in Korea and China [12, 13]. The leaves of this plant have been used for the treatment of lung and stomach disease, inflammatory problem, diabetes mellitus, and skin diseases [14]. Phytochemical studies with *E. japonica* have shown that the main constituents are quercetin, ursolic acid, oleanolic acid, tannin, chlorogenic acid, and caffeoylquinic acid [15, 16]. These compounds have been reported to be biologically active exhibiting anti-inflammatory, antiviral, antioxidant, cytotoxic, antimutagenic, anti-tumor, and hypoglycemic properties [17–19].

To date, various studies have been conducted to evaluate the different medicinal properties of *E. japonica* leaf extracts. There are, however, limited reports on the anti-inflammatory and anti-melanogenic activities of extracts of *E. japonica* leaf. In addition, the biological activities of 5% ethanol extract of *Eriobotrya japonica* leaf are almost unknown. In this study, the anti-oxidant, anti-inflammatory, and anti-melanogenic activities of *Eriobotrya japonica* leaf ethanol extract (EJEE) were evaluated different various in vitro models. In addition, for safety evaluation as cosmetic ingredient, skin and eye irritation test were performed using animal alternative tests. The animal alternative test was performed according to Organisation for Economic Cooperation and Development (OECD) guidelines or international validation study guideline.

## Results

### High-performance liquid chromatography (HPLC) analysis

The quantitative determination of constituents in the sample was performed with the help of a calibration curve. The HPLC analysis confirmed the presence of quercetin (71 mg/100 g of dried EJEE), which was previously identified [20] as a major constituent of the EJEE.

### Estimation of free radical-scavenging activity by 2,2-diphenyl-1-picrylhydrazyl (DPPH) assay

**Fig. 1:**
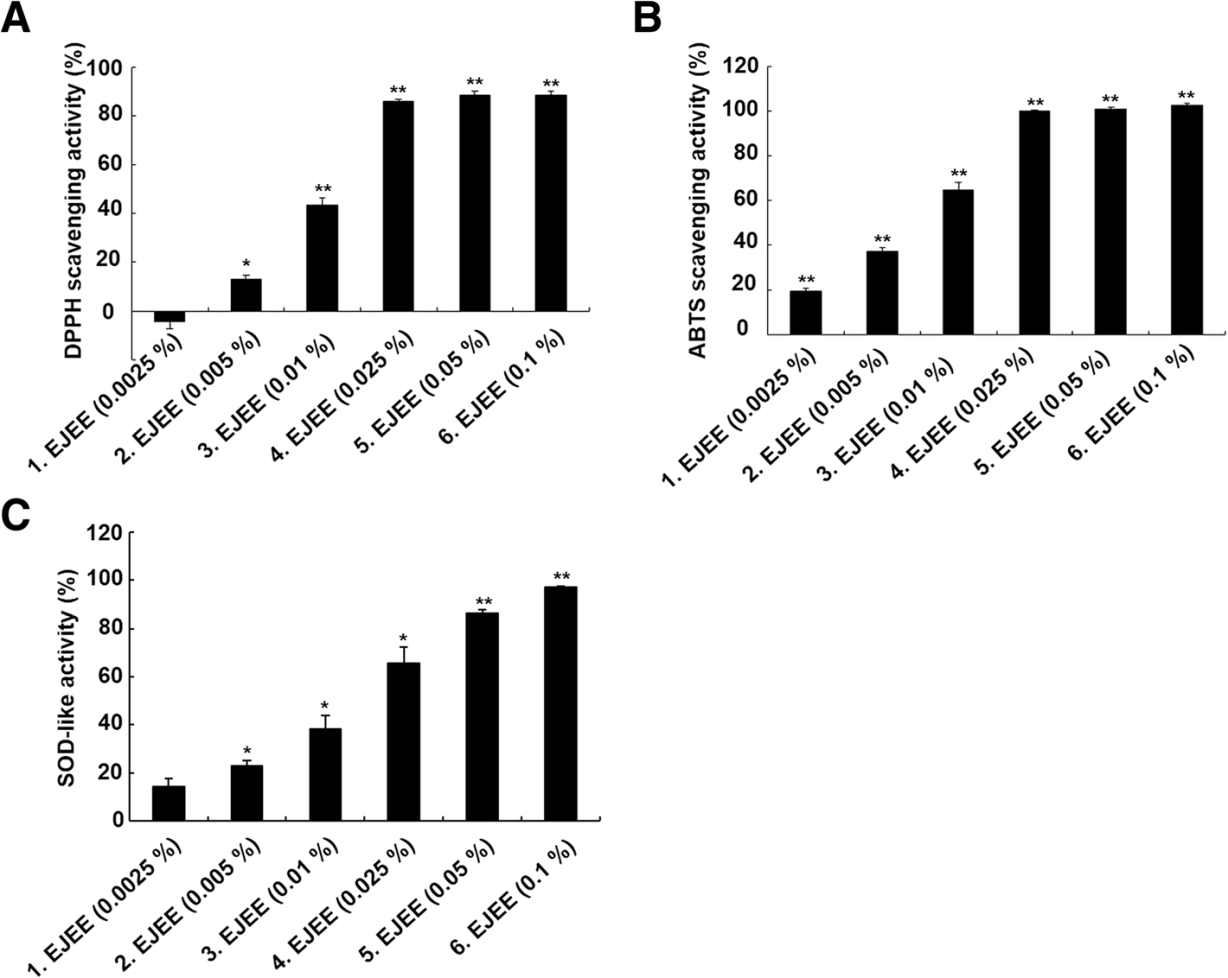
Anti-oxidant activity of EJEE in DPPH assay (**a**), ABTS assay (**b**), and SOD assay (**c**). Free radical scavenging activity and SOD-like activity were determined as described before. The values represent as the means ± SD of three independent experiments. ^*^p < 0.05 and ^**^p < 0.01 as compared to the non-treated sample

To evaluate the anti-oxidant effects of EJEE, the DPPH assay was performed for determination of the free radical scavenging activity. The anti-oxidant activity of EJEE was found to be concentration dependent (Fig. 1a). DPPH free radical scavenging activity was found to be − 4.64 ± 2.70, 13.14 ± 1.33, 43.33 ± 3.08, 85.96 ± 0.86, 88.65 ± 1.51, and 88.61 ± 1.35% at concentrations of 0.0025, 0.005, 0.01, 0.025, 0.05, and 0.1% (w/v), respectively. The extract exhibited a good dose-dependent activity with 86% scavenging capacity at a concentration of 0.025% (w/v).

### Estimation of free radical-scavenging activity by 2,2′-azino-bis(3-ethylbenzothiazolin-6-sulfonic acid (ABTS) assay

To analyze the anti-oxidant effect of EJEE, the ABTS assay was performed for determination of the free radical scavenging activity. ABTS free radical scavenging activity by EJEE was found to be 19.45 ± 1.24, 37.11 ± 1.58, 64.67 ± 3.51, 99.84 ± 0.39, 100.81 ± 0.75, and 102.42 ± 0.96% at concentrations of 0.0025, 0.005, 0.01, 0.025, 0.05, and 0.1% (w/v), respectively (Fig. 1b). In the ABTS assay, the extract exhibited a good dose-dependent activity with an IC_50_ scavenging capacity at a concentration of 0.01% (w/v).

### Estimation of anti-oxidant effect by superoxide dismutase (SOD) assay

To estimate the anti-oxidant activity of EJEE, the SOD assay was performed. The SOD-like activity by EJEE was found to be 14.19 ± 3.52, 22.80 ± 2.40, 38.39 ± 5.56, 65.78 ± 6.75, 86.31 ± 1.26, and 97.24 ± 0.38% at concentrations of 0.0025, 0.005, 0.01, 0.025, 0.05, and 0.1% (w/v), respectively (Fig. 1c). The extract exhibited a good dose-dependent activity with 50% SOD-like activity at a concentration of 0.025% (w/v).

### Estimation of anti-oxidant effect by total polyphenolic content (TPC) determination assay

To estimate the anti-oxidant activity of EJEE, the TPC determination assay was performed. The TPC was 88.68 ± 9.00 mg tannic acid equivalent (TAE)/g of EJEE (Table 1).

**Table 1:** Results of the total polyphenolic compounds amount in EJEE in different concentrations

Substance	OD_450_	Total polyphenol amount (mg/g)
EJEE	0.089 ± 0.009	88.68 ± 9.00

The values represent as the means ± standard deviation of triplet treatments

*EJEE Eriobotrya japonica* leaf ethanol extract

### Estimation of anti-inflammatory activity by NO assay

**Fig. 2:**
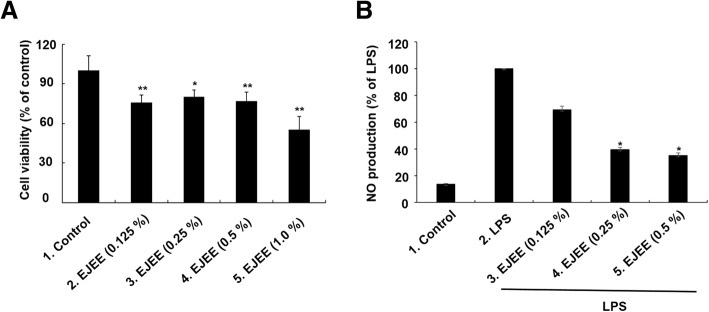
The effects of EJEE on cell viability (**a**) and LPS-induced NO production (**b**) in Raw 264.7 cells. The cells were treated different concentrations (0.125, 0.25, 0.5, and 1.0%) of EJEE for 24 h. The basal values (control) were obtained in the absence of EJEE. ^*^p < 0.05 and ^**^p < 0.01 as compared to the control group (**a**). The cells were treated with LPS 1 μg/mL and different concentrations (0.125, 0.25, and 0.5%) of EJEE for 24 h. The basal values (control) were obtained in the absence of LPS and EJEE. ^*^p < 0.05 as compared to the LPS-treated group (**b**)

To estimate the potential anti-inflammatory activity of EJEE, RAW 264.7 macrophage cells were used, which can produce NO after stimulation with lipopolysaccharide (LPS, 1 μg/mL). In order to exclude the possibility that the inhibitory effect of EJEE on NO production was due to the cytotoxic effects of EJEE in itself, cell viability test using 3-(4,5-dimethylthiazol-2-yl)-2,5-diphenyl-tetrazolium bromide (MTT) assay was performed. In the RAW 264.7 cells, the cell viability (% of control) upon treatment with EJEE was found to be 76.03 ± 5.44, 80.39 ± 4.84, 76.66 ± 7.24, and 54.98 ± 10.46 at concentrations of 0.125, 0.25, 0.5, and 1.0% (w/v), respectively (Fig. 2a). The cell viability of the RAW 264.7 cells was not affected by EJEE up to a concentration of 0.5%. Based on this result, the highest concentration of EJEE was determined to be 0.5% (w/v). The present study demonstrated that EJEE has an inhibitory effect on the production of NO in LPS (1 μg/ml)-stimulated RAW 264.7 macrophage cells at a concentration of > 0.25% (w/v). The inhibitory effects on NO production by EJEE in the LPS-induced RAW 264.7 cells were found to be 69.24 ± 2.75, 39.54 ± 1.70, and 35.25 ± 1.81% compared to the LPS-treated group at concentrations of 0.125, 0.25, and 0.5% (w/v), respectively. (Fig. 2b).

### Estimation of anti-inflammatory activity by TNF-α measurement

To analyze the potential anti-inflammatory activity of EJEE, RAW 264.7 macrophages were used, which can produce TNF-α after stimulation with LPS (1 μg/mL). Based on the cytotoxicity test results, the highest concentration of EJEE was determined to be 0.5% (w/v). The present study demonstrated that EJEE has a little inhibitory effect on the expression of TNF-α in LPS (1 μg/ml)-stimulated RAW 264.7 macrophage cells at a concentration of 0.5% (w/v). The effects on TNF-α release by EJEE in the LPS-induced RAW 264.7 cell were found to be 889.74 ± 11.96, 888.39 ± 7.16, and 839.43 ± 6.81 pg/mL at concentrations of 0.125, 0.25, and 0.5% (w/v), respectively. (Table 2).

**Table 2:** Results of the TNF-α production in EJEE in different concentrations

Substance	EJEE concentration (%)	TNF-α (pg/mL)
–	–	280.36 ± 22.09
LPS	–	907.97 ± 7.37
EJEE + LPS	0.125	889.74 ± 11.96
	0.25	888.39 ± 7.16
	0.5	839.43^*^ ± 6.81

The values represent as the means ± standard deviation of triplet treatments

Significant differences were compared with LPS, ^*^ *P* < 0.05

*EJEE Eriobotrya japonica* leaf ethanol extract, *LPS* lipopolysaccharide, and *TNF-α* tumor necrosis factor-α

### Estimation of anti-melanogenic effect by melanogenesis inhibition test

**Fig. 3:**
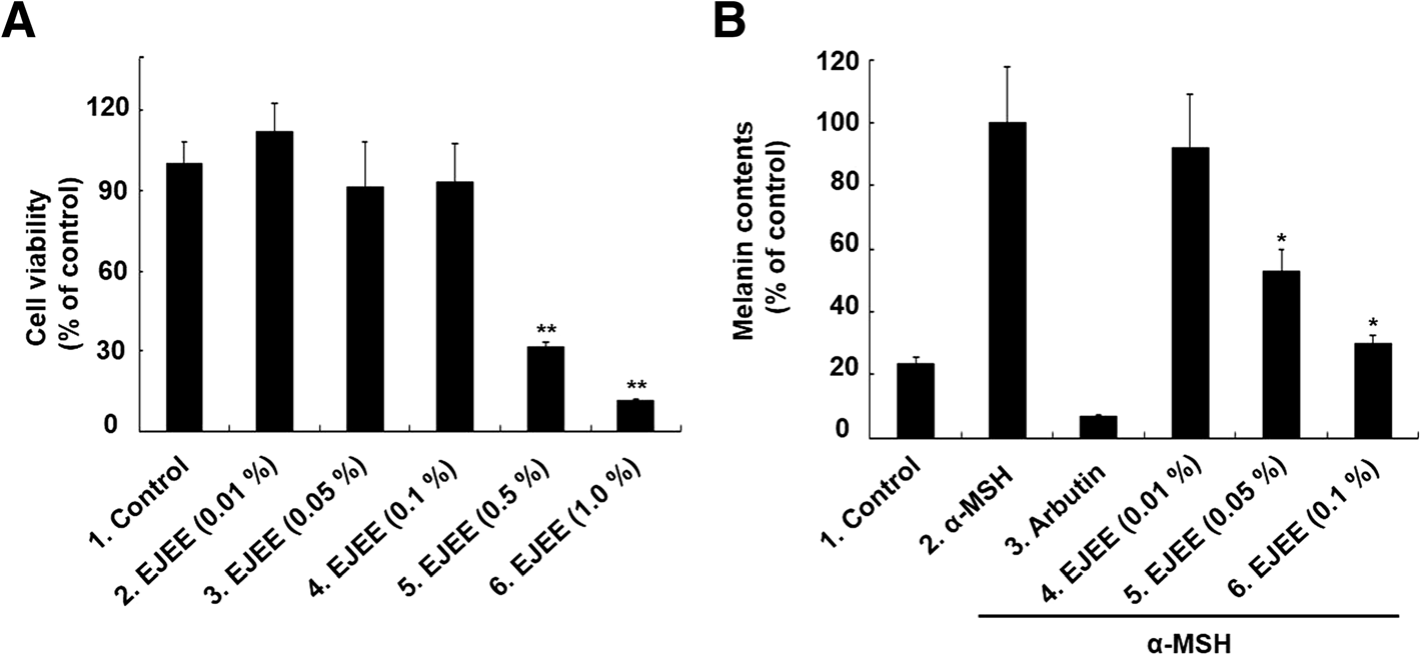
The effects of EJEE on cell viability (**a**) and melanin contents (**b**). The cells were treated different concentrations (0.01, 0.05, 0.1, 0.5, and 1.0%) of EJEE for 24 h. The basal values (control) were obtained in the absence of EJEE. ^**^p < 0.01 as compared to the control group (**a**). The cells were treated with α-MSH (100 nM) and different concentrations (0.01, 0.05, and 0.1%) of EJEE for 72 h. Arbutin (10 mM) was used as a positive control. The values represent as the means ± SD of three independent experiments. ^*^p < 0.05 as compared to the α-MSH-treated group (**b**)

To analyze the anti-melanogenic effects of EJEE, B16F1 mouse melanoma cells were used, which can produce melanin after stimulation with α-MSH (100 nM). In B16F1 cells, cell viability (% of control) by EJEE was found to be 112.23 ± 10.63, 91.38 ± 16.99, 93.02 ± 14.63, 31.51 ± 1.53, and 11.36 ± 0.75 at concentrations of 0.01, 0.05, 0.1, 0.5, and 1.0% (w/v), respectively (Fig. 3a). The cell viability of the B16F1 cells was not affected by EJEE up to a concentration of 0.1% (w/v). Based on this result, the highest concentration of EJEE was determined to be 0.1% (w/v). The melanin content compared to the α-MSH induced melanin amount was found to be 92.3 ± 17.1, 52.9 ± 6.9, and 29.7 ± 2.8% at concentrations of 0.01, 0.05, and 0.1%, respectively (Fig. 3b). The melanin content was significantly inhibited by EJEE at concentrations of 0.05 and 0.1% (w/v).

### Estimation of skin irritation by reconstructed human epidermis (RHE) model test

The result of skin irritation test using SKINETHIC™ RHE model was given in Table 3. EJEE was evaluated as a non-irritant substance corresponding to ‘No Category’ based on the Globally Harmonized System of Classification and Labelling of Chemicals [23] because cell viability was 98.9 ± 2.0% compared with negative control.

**Table 3:** Results of the skin irritation test in EJEE

Substance	Concentration (%)	OD_570_	Cell viability (%)
PBS	–	2.379 ± 0.037	100.0 ± 1.6
SDS	5	0.022 ± 0.006	0.9 ± 0.2
EJEE	neat	2.353 ± 0.048	98.9 ± 2.0

The values represent as the means ± standard deviation of three RHE wells

*EJEE Eriobotrya japonica* leaf ethanol extract, *PBS* phosphate buffered saline, *SDS* sodium dodecyl sulfate

### Estimation of eye irritation by bovine corneal opacity and permeability (BCOP) assay

In BCOP assay, both EJEE-treated group and negative control group were not observed opacity and permeability increase grossly. EJEE is evaluated as a non-irritant substance corresponding to ‘No Category’ based on the Globally Harmonized System of Classification and Labelling of Chemicals [24] because In vitro irritancy score (IVIS) was 2.2 ± 0.5 compared with the negative control (Table 4).

**Table 4:** Results of the eye irritation test using BCOP assay of EJEE

Substance	Opacity	Permeability	IVIS
NC	−0.8 ± 0.6	0.003 ± 0.004	−0.7 ± 0.7
PC	44.4 ± 1.9	3.081 ± 0.023	90.6 ± 2.1
EJEE	2.2 ± 0.5	−0.001 ± 0.003	2.2 ± 0.5

The values represent as the means ± standard deviation of three corneas

*NC* sodium chloride 0.9% solution, *PC* imidazole

*EJEE Eriobotrya japonica* leaf ethanol extract, *IVIS* in vitro irritancy score

### Estimation of eye irritation by hen’s egg test-chorioallantoic membrane (HET-CAM) assay

As a result of gross evaluations, both EJEE-treated group and negative control group were not observed any response such as hemorrhage, coagulation and lysis. The EJEE did not cause significant ocular damage and was a non-irritating substance in HET-CAM assay because S score was calculated from 0 scores (Table 5).

**Table 5:** Results of the eye irritation test using HET-CAM assay of EJEE

Substance	Concentration (%)	Number of Eggs	[S] score
NC	–	6	0
PC	1	6	13
EJEE	20	6	0

NC: sodium chloride 0.9% solution; PC: sodium dodecyl sulfate; [S]: scores are presented as the sum of six eggs

EJEE, *Eriobotrya japonica* leaf ethanol extract

## Discussion

Phenolic compounds and flavonoids exhibit various beneficial effects on the skin such as anti-aging, anti-inflammatory, and anti-bacterial properties. According to a previous report by Pande and Akoh [26], the extracts of *Eriobotrya japonica* leaf contain flavonoids (quercetin, catechin, and epicatechin), and phenolic acids (gallic acid, ellagic acid, caffeic acid, and *p*-coumaric acid). Ahumada et al. [27] also reported that the content of quercetin derivatives in the leaf extracts was 26–99 mg/100 g. In this study, the quercetin content of EJEE was found to be 71.0 ± 16.1 mg/100 g and the total polyphenol content was determined to be 88.68 mg TAE/g. In this study, the anti-oxidant, anti-inflammatory, and anti-melanogenic activities of EJEE were evaluated using various in vitro models. In addition, we investigated the potential irritation of EJEE to skin and eye using animal alternative tests.

According to a number of previous studies, the ethanol extract of *Eriobotrya japonica* leaf exhibits anti-oxidant activity, such as DPPH free radical-scavenging activity and SOD-like activity [28, 29]. The biological activity of the 5% ethanol extract of *Eriobotrya japonica* leaf was, however, unknown. In this study, EJEE (0.1% w/v) showed a free radical-scavenging ability of 88.61% (DPPH assay), and a free radical-scavenging ability of 102.42% (ABTS assay). In addition, EJEE (0.1%) showed 97.24% SOD-like activity. The increase in ROS production causes oxidative stress, which is the main characteristic of aging-related degenerative disease [30]. Oxidative stress is involved in the pathogenesis of skin and causes changes in melanogenesis [31]. In plants, the anti-oxidant activity of some natural compounds, such as vitamins, minerals, and polyphenols inhibits the production of free radicals and is often used as a free radical scavenger [32, 33]. Overall, these results indicate that EJEE has an anti-oxidant effect and a strong free radical scavenging activity.

In this study, we have evaluated whether EJEE could control one of the ROS-mediated inflammatory response, such as LPS-induced stimulation of inflammatory mechanism in macrophages. NO is recognized as a mediator and regulator of the several inflammatory responses and produces reactive nitrogen species that react with molecular oxygen and superoxide anions and alter many cellular functions. NO is generated by iNOS as an inflammatory response, and ROS produced by oxidative stress is synthesized by the activated inflammatory cells [34]. TNF-α was originally described as a circulating factor that could cause tumor necrosis. It has been, however, identified as a key regulator of the inflammatory response, and has been implicated in a variety of diseases, such as inflammatory, infectious, and malignant disease [35]. According to a report by Cha et al. [36], extract of *Eriobotrya japonica* leaf exerts inhibitory effects on the different inflammatory mediators including NO, iNOS, cyclooxygnase-2, TNF-α, and interleukin (IL)-6 by the attenuation of nuclear factor-κB (NF-κB) into the nucleus. Thus, inhibition of NO and TNF-α may be considered a promising strategy to prevent inflammation. In this study, NO production and TNF-α expression were significantly reduced by 0.5% (w/v) of EJEE compared to LPS. In a previous study, the anti-inflammatory effects of loquat tea extract were demonstrated to be mediated by the inhibition of iNOS, NO, IL-6, and TNF-α production, and downregulation of the transforming growth factor-β-activated kinase-mediated mitogen-activated protein kinases and NF-κB pathways in the mouse macrophage-like RAW 264.7 cells [37]. Further, extract of *Eriobotrya japonica* leaf was found to modulate the production of pro-inflammatory cytokines, such as TNF-α, IL-6, and IL-8 in mast cells [18], and also reduced the production of TNF-α in phorbol 12-myristate 13-acetate and A23187-stimulated human mast cells [16]. Therefore, EJEE exerts anti-inflammatory effects by inhibiting NO and TNF-α activity.

We investigated whether EJEE can regulate melanin production because melanin plays an essential role against UV-induced ROS production, EJEE has demonstrated potent anti-oxidant and anti-inflammatory activities [38, 39]. Recent studies have shown that hyperpigmentation is caused by various inflammatory skin disorders, such as eczema, and dermatitis [8, 9, 40, 41]. In this study, melanin content was significantly inhibited by EJEE at concentrations of 0.05 and 0.1%. Recently, Tan et al. [42] reported that the methanolic extract of *Eriobotrya japonica* leaf exhibits a significant dose-dependent inhibition of melanogenesis in B16 melanoma cells. In addition, for whitening effects, 30% ethanol and 70% ethanol extracts of *Eriobotrya japonica* leaf showed mushroom tyrosinase inhibitory activity at a concentration of 5% [43]. Collectively, EJEE exerts anti-melanogenic effects by its anti-oxidant and anti-inflammatory activities.

*Eriobotrya japonica* leaf exhibits diverse pharmacological properties, but the potential toxic effects of the extract still remain unclear in the skin and eye [37]. Therefore, we performed skin irritation test using RHE model, and performed eye irritation test using BCOP and HET-CAM assay. The skin irritation test using the RHE model was a widely accepted skin irritation assay for cosmetics to identify substances that can consider as an irritant or non-irritant substance based on the UN GHS Category [23]. The present study showed that no toxicity of skin irritation to EJEE in RHE model. Also, we evaluated the eye irritation of EJEE by combining the BCOP and HET-CAM assay to detect entire ocular irritation. The purpose of the BCOP assay is to identify agents that cause corneal damage and HET-CAM assay is to identify substances that cause conjunctival irritation [44]. This is a widely accepted eye irritation assay for cosmetics to identify substances that can consider as an irritant or non-irritant substance based on the UN GHS Category [24]. Our results indicated that EJEE was no eye irritation at the suggested concentrations.

## Conclusions

The results of this study indicate that EJEE can protect the human skin against oxidative stress and inflammation due to the high polyphenol and quercetin content. These compounds suppress NO production and TNF-α expression and also exert free-radical scavenging activity and melanin synthesis inhibition. Furthermore, EJEE did not cause irritation of skin and eye by animal alternative tests. Therefore, EJEE has demonstrated good anti-oxidant, anti-inflammatory, and anti-melanogenesis activities, while it has no risk of skin and eye. These results are expected to provide important information on skin improvement by EJEE.

## Methods

### Preparation of EJEE

EJEE used in this study was purchased by the Korea INS Pharm Research Institute (Hwasun, Korea). *Eriobotrya japonica* leaves were extracted by reflux method using 5% ethanol for 4 h at 100 °C. The extracts were filtered, concentrated under decompression, and spray-dried to obtain the final extract [15]. The required drug concentrations were prepared by dilution.

### HPLC analysis

The chromatographic separations were performed using an HPLC (Agilent Technologies 1200 series, Santa Clara, CA, USA) equipped with Agilent 1200 series binary pumps, a diode array detector, vacuum degasser, and Rheodyne injection valve. The samples were separated by a C_18_-UG120 column (250 × 4.6 mm, 5 μm, Shiseido, Tokyo, Japan). The chromatography conditions were: temperature, 35 °C, wavelength 370 nm; injection volume, 10 μL: and flow rate 0.8 mL/min. The mobile phase comprised of water and 5% acetic acid (*aq.*)**–**acetonitrile (40:30:30, v/v/v) for quercetin estimation. Furthermore, the mobile phase was filtered through a 0.45-μm polytetrafluoroethylene membrane filter. The chromatographic separation of quercetin was achieved within 20 min. Previous studies have reported that dietary flavonoids such as quercetin exhibit potent antioxidant, immunomodulatory, anti-inflammatory, anti-atherosclerotic, and antiplatelet properties [20, 21]. Thus, in the present study, quercetin, one of the active ingredients, was designated as a standard reference to identify and quantify the major component of EJEE [20].

### Cell culture

Normal murine macrophage (RAW 264.7 cell; ATCC, USA) and mouse melanoma (B16F1 cell; ATCC, USA) cells were maintained in Dulbecco’s Modified Eagle’s Medium (DMEM) supplemented with 100 U/mL of penicillin, 100 g/mL of streptomycin, and 10% fetal bovine serum (FBS; Gibco BRL Life Technologies Inc., Gaithersburg, MD, USA). The cells were grown at 37 °C in a humidified chamber containing 5% CO_2_. The cells were incubated with EJEE in different concentrations or with a positive control and stimulated with LPS (Sigma Aldrich) or α-melanocyte stimulating hormone (α-MSH; Sigma Aldrich) for evaluation of the anti-inflammatory or anti-melanogenic activity.

### Cell viability test

The viability of RAW 264.7 and B16F1 cells treated with EJEE was determined by MTT assay, measuring absorbance at 570 nm wavelength at which the blue-colored formazan crystal concentration could be determined. Briefly, the B16F1 (1 × 10^4^ cells/well) and RAW 264.7 cells (5 × 10^4^ cells/well) were cultured in 96-well plates and treated with different doses of EJEE for 24 h. The cells were then exposed to MTT (Sigma Aldrich) solution (5 mg/mL in DPBS) for 3 h, after which all media were removed and isopropanol (Junsei, Tokyo, Japan) was added to each well. The amount of solubilized formazan product, indicating the number of viable cells, was determined by absorbance using EPOCH microplate reader (BioTek, USA) at 570 nm wavelength. The MTT assay was conducted three times, each time with three independent observations. The average result was noted before the anti-inflammatory activity test and melanogenesis inhibition test was conducted.

### Measurement of the free radical-scavenging activity by DPPH assay

To determine free radical scavenging activity of EJEE in vitro, cell-free chemically based DPPH assay was performed using EJEE in different concentrations (0.1, 0.05, 0.025, 0.01, 0.005, and 0.0025%, w/v), and synthetic anti-oxidant L-ascorbic acid (Sigma Aldrich) as a positive control (0.002, 0.015, 0.001, and 0.0005%, w/v). One milliliter of the test substance (0.1, 0.05, 0.025, 0.01, 0.005, and 0.0025%, w/v) was mixed with 1 mL of DPPH reagent (Sigma Aldrich; 0.002% (w/v) methanol solution). After incubation in the dark at room temperature for 30 min, the absorbance was determined on an EPOCH microplate reader at 517 nm wavelength. The tests were performed in triplicate. DPPH radical scavenger activity was calculated as follows: DPPH radical scavenger activity (%) = (A – B) / A × 100 (%), where A is the UV absorbance of the control (containing all reagents except the extract), and B is the UV absorbance of test sample.

### Measurement of the free radical-scavenging activity by ABTS assay

The radical cation (ABTS^·+^; Sigma Aldrich) was prepared by mixing 7.2 mM ABTS^·+^ solution with 2.4 mM potassium persulfate (1:1, v/v). This mixture was stored for 16 h in the dark until the reaction was complete. EJEE was prepared at different concentrations (0.1, 0.05, 0.025, 0.01, 0.005, and 0.0025%, w/v). The synthetic anti-oxidant L-ascorbic acid (positive control) was used in the following concentrations: 0.0025, 0.001, 0.0005, and 0.0001% (w/v). The ABTS^·+^ solution was diluted with ethanol until its absorbance was 1.00 ± 0.05 at 734 nm wavelength. The photometric assay was performed on 900 μL of diluted ABTS^·+^ solution and 100 μL of test samples which were mixed for 30 min in a dark. The absorbance was determined in an EPOCH microplate reader at 734 nm wavelength. The tests were carried out in triplicate. The anti-oxidant activity of EJEE was calculated as follows: ABTS radical scavenger activity (%) = [(B – A) / B] × 100 (%), where A is the UV absorbance of test sample, and B is the UV absorbance of control (ABTS^·+^ solution which diluted with ethanol).

### Evaluation of the anti-oxidant activity by SOD-like activity assay

In order to determine the SOD-like activity of EJEE, SOD activity was estimated using a SOD determination kit (Sigma-Aldrich) following the manufacturer’s instructions. EJEE was prepared at different concentrations (0.1, 0.05, 0.025, 0.01, 0.005, and 0.0025% w/v). The synthetic anti-oxidant L-ascorbic acid (positive control) was used in the following concentrations: 0.0025, 0.001, 0.0005, and 0.0001% (w/v). Two hundred microliters of the sample was mixed with 2.6 mL of Tris-HCL buffer (pH 8.5) and incubated at 25 °C for 10 min, after which 0.1 mL of 1 M HCL was added to stop the reaction. The absorbance was determined in an EPOCH microplate reader at 450 nm wavelength. The tests were carried out in triplicate. The SOD activity of the test material was calculated as follows: SOD-like activity (%) = [(S1 – S3) – (SS – S2) / (S1 – S3)] × 100 (%), where S1 is the UV absorbance of blank 1 (distill water + WST working solution + enzyme working solution), S2 is the UV absorbance of blank 2 (sample + WST working solution + dilution buffer), S3 is the UV absorbance of blank 3 (distill water + WST working solution + dilution buffer), SS is the UV absorbance of the test sample (sample + WST working solution + enzyme working solution).

### Evaluation of the anti-oxidant activity by the TPC determination assay

The TPC in EJEE was determined by a modified Folin-Ciocalteu method [22]. Briefly, EJEE was prepared at 10 mg/mL (w/v) in distill water. Twenty microliters of EJEE solution was mixed with 100 μL of 1 M Folin-Ciocalteu phenol reagent (Sigma Aldrich). Then, this mixture was kept standing for 3 min at room temperature, following which, 80 μL of 10% Na_2_CO_3_ solution was added. The mixture was again incubated for 1 h at room temperature. The absorbance was determined by EPOCH microplate reader at 725 nm wavelength. The tests were carried out in triplicate. A calibration curve was prepared with tannic acid (50–500 μg/mL).

### Evaluation of the anti-inflammatory activity by NO assay

In order to determine the concentration of NO, nitrite (NO^2−^) was estimated using a nitrite/nitrate assay kit (Sigma-Aldrich) following the manufacturer’s instructions. After pre-incubation of the RAW 264.7 cells (5 × 10^4^ cells/well) for 24 h, the cells were incubated with 0.125, 0.25, and 0.5% (w/v) of EJEE with LPS (1 μg/mL) for 24 h. One hundred microliters of the supernatant from each well of the cell culture plates were transferred to 96-well microplates, and the supernatant was mixed with an equal volume of Griess reagent at room temperature. The absorbance was determined by EPOCH microplate reader at 550 nm wavelength. The percentage of inhibition was calculated based on the ability of EJEE to inhibit nitric oxide formation by the cells compared to the control (cells in media without EJEE containing LPS). The tests were carried out in triplicate.

### Evaluation of the anti-inflammatory activity by TNF-α measurement

In order to evaluate the anti-inflammatory effect of EJEE, TNF-α levels were estimated using a mouse TNF-α enzyme-linked immunosorbent assay kit (R&D SYSTEMS, USA). Briefly, after pre-incubation of the RAW 264.7 cells (5 × 10^4^ cells/well) for 24 h, the cells were treated with 0.125, 0.25, and 0.5% of EJEE with LPS (1 μg/ml) for 24 h. The supernatant from each well of the cell culture plates was transferred to 96-well microplates, and subsequent steps were performed following the manufacturer’s instructions. The absorbance was determined by EPOCH microplate reader at 560 nm wavelength. The tests were carried out in triplicate.

### Evaluation of the anti-melanogenic effect by melanogenesis inhibition test

In order to evaluate the anti-melanogenic effects of EJEE, the melanin content was measured by melanogenesis inhibition test. Briefly, B16F1 cells were cultured at 1 × 10^5^ cells in 10% FBS-DMEM at 37 °C in the presence of 5% CO_2_. After 24 h, the cells were treated with various concentrations of EJEE (1.0, 0.5, 0.1, 0.05, and 0.01%, w/v). Ten millimolar Arbutin (Sigma Aldrich) as used as a positive control. One hundred nanomolar α-MSH (Sigma Aldrich) as a melanin production inducer was added to the medium for 72 h. The cells were harvested by trypsinization. After washing DPBS for two times, the samples were dissolved in 1 N NaOH (Sigma Aldrich) containing 10% dimethyl sulfoxide (DMSO, Sigma Aldrich). The samples were then heated at 60 °C for 20 min. The amount of melanin was determined based using an EPOCH microplate reader at 490 nm wavelength. The tests were carried out in triplicate.

### Skin irritation test by RHE model

The skin irritation test was performed according to the OECD Test Guideline 439 [23]. The SKINETHIC™ RHE model (EPISKIN, France) was used in this study. Each insert containing the RHE tissue was removed from the agarose gel and transferred to the 6-well plate with 1 mL of growth media (EPISKIN, France). The plate was incubated at 37 °C, 5% CO_2_ incubator for more than 2 h for stability. And then, 10 μL of the sterilized distilled water was topically applied to the upper epithelial surface of the tissue and then 16 mg of EJEE was applied. EJEE-treated RHE tissues was kept for 42 min exposure at room temperature. After 42 min, EJEE-treated RHE tissues were washed with PBS. The RHE tissue surface was rinsed 25 times (1 mL/times) to remove all residual test samples. Washed RHE tissue was transferred to the new 6-well plate with 2 mL of growth media. The plate was incubated at 37 °C, 5% CO_2_ incubator for 42 h. After removing all medium, RHE tissues were transferred to the 24-well plate with 0.3 mL of MTT solution (1 mg/mL). The plate was incubated in the 37 °C, 5% CO_2_ incubator for 3 h. The RHE tissues were transferred to 24 well-plate with 0.8 mL of isopropanol and added 0.7 mL isopropanol inside insert. After covering the 24-well plate with aluminum foil, 24-well plate was extracted formazan for 2 h at room temperature with gentle shaking on a plate shaker. Formazan solution was transferred to the 96-well plate. The absorbance was determined by EPOCH microplate reader at 570 nm wavelength.

### Eye irritation test by BCOP assay

The BCOP assay was performed according to the OECD Test Guideline 437 [24]. Briefly, bovine eyes were collected at a slaughterhouse as soon as possible after death and completely immersed in Hanks’ Balanced Salt Solution (HBSS; Life Technologies, USA). To minimize contamination during transport, the eyes were kept on wet ice during the collection and transportation and in medium HBSS containing penicillin at 100 IU/mL and streptomycin at 100 μg/ml. Isolated corneas were mounted in holders and filled to excess with pre-warmed Eagle’s Minimum Essential Medium (EMEM without phenol red; Life Technologies, USA). The holders were then equilibrated at 32 °C for 1 h. The corneas were exposed to EJEE at 20% (w/v) prepared in 0.9% NaCl solution (Sigma Aldrich). After 4 h of exposure, EJEE was removed and the corneas were washed using EMEM (with phenol red; Life Technologies, USA) at least three times to eliminate the test sample. The effects of EJEE to cornea were measured by decreased light transmission (corneal opacity) using an OP3.0 opacitometer (BASF, Germany) and increased passage of sodium fluorescein dye (permeability). One milliliter of sodium fluorescein solution (5 mg/mL; Sigma Aldrich) was added to the anterior chamber of the corneal holder while the posterior chamber was filled with fresh EMEM (w/o phenol red). The holders were then incubated for 90 min at 32 °C. The amount of sodium fluorescein that crossed into the posterior chamber was quantitatively determined by EPOCH microplate reader at 490 nm wavelength. The opacity and permeability assessments of the cornea after exposure to the EJEE were combined to derive an IVIS, used to classify the irritancy level of each exposed group (Table 6).

**Table 6:** The opacity and permeability assessments of the cornea

In vitro irritancy score (IVIS)	UN GHS
≤ 3	No Category
> 3; ≤ 55	No prediction can be made
> 55	Category 1

*IVIS* = mean opacity value + (15 × mean permeability value)

*UN GHS* United nations globally harmonized system of classification and labelling of chemicals

### Eye irritation test by HET-CAM assay

The HET-CAM assay was performed according to the HET-CAM BRD [25]. All eggs were incubated for 9 days. And then, they were selected from fresh fertilized 50–60 g eggs on day 10; their blunt ends of eggs were checked by illuminated with a candling lamp and then unfertilized eggs discarded. The part of air space on selected eggs was marked by permanent pen. The egg shell was pared along the marked line with forceps and visible white egg membrane was moistened with 0.9% NaCl solution (Sigma Aldrich). Before application of the EJEE, the 0.9% NaCl solution was poured off and the moistened egg membrane was carefully removed with forceps without injuring any underlying blood vessels. The chorioallantoic membrane (CAM) and its vascular system was exposed and assessed in terms of its suitability for use. Only eggs on which a distinct fine vascular system was recognized on the CAM used for testing. Test method was selected end point assessment by test sample appearance. The test sample was applied to six prepared eggs in a dose 0.3 mL onto the CAM surface for 30 s. After 30 s of treatment time, test substance was carefully rinsed on the CAM with 0.9% NaCl solution. By analogy to reference substance testing, possible reactions were recorded semi-quantitatively by type (Table 7). The test substance was classified by the sum of all standard end point scores of reaction according to the classification scheme (Table 8).

**Table 7:** The end point scores and possible reactions by analogy to reference substance testing

End point score	Reaction	Reference substance
0	No reaction	–
1	Slight reaction	Texapon ASV 50 (0.5%)
2	Moderate reaction	Texapon ASV 50 (1%)
3	Severe reaction	Texapon ASV 50 (5%)

**Table 8:** The classification scheme of test substance by sum of all standard end point scores of reaction

[S] score	Irritation
S < 6	Nonirritant or Slightly irritating
6 ≤ S ≤ 12	Moderately irritating
12 < S < 16	Irritating
16 ≤ S	Severely irritating

### Statistical analysis

The data of the three experiments performed in triplicates were represented as means ± standard deviation (SD). Statistical analysis was performed by Student’s *t*-test using SPSS version 19.0 (SPSS, Inc., Chicago, IL, USA). Differences were considered significant at ^*^ *p* < 0.05 and ^**^ *p* < 0.01.

## Abbreviations

ABTS: 2,2′-Azino-bis(3-ethylbenzothiazolin-6-sulfonic acid
BCOP: Bovine corneal opacity and permeability
CAM: Chorioallantoic membrane
DMEM: Dulbecco’s Modified Eagle’s Medium
DMSO: Dimethyl sulfoxide
DPPH: 2,2-Diphenyl-1-picrylhydrazyl
EJEE: *Eriobotrya japonica* leaf ethanol extract
EMEM: Eagle’s Minimum Essential Medium
FBS: Fetal bovine serum
HBSS: Hanks’ Balanced Salt Solution
HET-CAM: Hen’s egg test-chorioallantoic membrane
HPLC: High-performance liquid chromatography
iNOS: Inducible nitric oxide synthase
IVIS: In vitro irritancy score
LPS: Lipopolysaccharide
MTT: 3-(4,5-Dimethylthiazol-2-yl)-2,5-diphenyl-tetrazolium bromide
NO: Nitric oxide
OECD: Organisation for Economic Cooperation and Development
RHE: Reconstructed human epidermis
ROS: Reactive oxygen species
SD: Standard deviation
SOD: Superoxide dismutase
TNF-α: Tumor necrosis factor-α
TPC: Total polyphenolic content
UV: Ultraviolet
α-MSH: α-Melanocyte stimulating hormone

## References

[1] Chen JS, Wei C, Marshall MR (1991). Inhibition mechanism of Kojic acid on polyphenol oxidase. J Agric Food Chem.

[2] Couteau C, Coiffard L (2016). Overview of skin whitening agents: drugs and cosmetic products. Cosmetics.

[3] Jin KS, Oh YN, Park JA, Lee JY, Jin SJ, Anti-Oxidant HSK (2012). Anti-Oxidant, anti-melanogenic, and anti-inflammatory activities of *Zanthoxylum schinifolium* extract and its solvent fractions. Korean J Microbiol Biotechnol.

[4] Kang MC, Lee JY, Ko RK, Kim HB, Hong SH, Kim GO (2009). Melanin inhibitory effect and anti-inflammatory effects of Dictyota corlacea extracts derived from adjacent sea of the Jeju island. Korean J Biotechnol Bioeng.

[5] Wang G, Chen K, Chen L, Hu C, Zhang D, Liu Y (2008). The involvement of the antioxidant system in protection of desert cyanobacterium Nostoc sp. against UV-B radiation and the effects of exogenous antioxidants. Ecotoxicol Environ Saf.

[6] Huang HC, Lien HM, Ke HJ, Chang LL, Chen CC, Chang TM (2012). Antioxidative characteristics of *Anisomeles indica* extract and inhibitory effect of Ovatodiolode on melanogenesis. Int J Mol Sci.

[7] Kawabata T, Cui MY, Hasegawa T, Takano F, Ohta T (2011). Anti-inflammatory and anti-melanogenic steroidal saponin glycosides from fenugreek (Trigonella foenum-graecum L.) seeds. Planta Med.

[8] Callender VD, St Surin-Lord S, Davis EC, Maclin M (2011). Postinflammatory hyperpigmentation: etiologic and therapeutic considerations. Am J Clin Dermatol.

[9] Panich U, Tangsupa-a-nan V, Onkoksoong T, Kongtaphan K, Kasetsinsombat K, Akarasereenont P (2011). Inhibition of UVA-mediated melanogenesis by ascorbic acid through modulation of antioxidant defense and nitric oxide system. Arch Pharm Res.

[10] Laskin JD, Rao NR, Punjabi CJ, Laskin DL, Synder R (1995). Distinct actions of benzene and its metabolites on nitric oxide production by bone marrow leukocytes. J Leukoc Biol.

[11] Kwamata H, Ochiai H, Mantani N, Terasawa K (2000). Enhanced expression of inducible nitric oxide synthasw by Juzen-taiho-to in LPS-activated RAW 264.7 cells, a murine macrophage cell line. Am J Chin Med.

[12] Matalka KZ, Abdulridha NA, Badr MM, Mansoor K, Qinna NA, Qadan F (2016). *Eriobotrya japonica* water extract characterization: an inducer of interferon-gamma production mainly by the JAK-STAT pathway. Molecules.

[13] Uto T, Sakamoto A, Tung NH, Fujiki T, Kishihara K, Oiso S, Kariyazono H, Morinaga O, Shoyama Y (2013). Anti-proliferative activities and apoptosis induction by triterpenes derived from *Eriobotrya japonica* in human leukemia cell lines. Int J Mol Sci.

[14] Alshaker HA, Qinna NA, Qadan F, Bustami M, Matalka KZ. *Eriobotrya japonica* hydrophilic extract modulates cytokines in normal tissues, in the tumor of meth-A-fibrosarcoma bearing mice, and enhances their survival time. BMC Complement Altern Med. 2011;11(9). 10.1186/1472-6882-11-9. 10.1186/1472-6882-11-9 PMC3045389 21294856

[15] Bae D, Kim J, Na JR, Kim Y, Lee JY, Kim S (2014). Anti-amnesic effect of *Eriobotrya japonica* leaf extract on scopolamine-induced memory impairment in rats. J Korean Soc Food Sci Nutr.

[16] Kim SH, Kwon YE, Park WH, Jeon H, Shin TY (2009). Effect of leaves of *Eriobotrya japonica* on anaphylactic allergic reaction and production of tumor necrosis factor-alpha. Immunopharmacol Immunotoxicol.

[17] Banno N, Akihisa T, Tokuda H, Yasukawa K, Taguchi Y, Akazawa H, Ukiya M, Kimura Y, Suzuki T, Nishino H (2005). Anti-inflammatory and antitumor-promoting effects of the triterpene acids from the leaves of *Eriobotrya japonica*. Biol Pharm Bull.

[18] Maher K, Yassine BA, Sofiane B (2015). Anti-inflammatory and antioxidant properties of *Eriobotrya japonica* leaves extracts. Afr Health Sci.

[19] Takashi K, Hiroyuki A, Keiichi T, Aranya M, Jiradej M, Takashi S, Toshihiro A (2011). 3-O-(E)-p-coumaroyl tormentic acid from *Eriobotrya japonica* leaves induces caspase-dependent apoptotic cell death in human leukemia cell line. Chem Pharm Bull.

[20] Jung HA, Park JC, Chung HY, Kim J, Choi JS (1999). Antioxidant flavonoids and chlorogenic acid from the leaves of *Eriobotrya japonica*. Arch Pharm Res.

[21] Oh Won Jun, Endale Mehari, Park Seung-Chun, Cho Jae Youl, Rhee Man Hee (2012). Dual Roles of Quercetin in Platelets: Phosphoinositide-3-Kinase and MAP Kinases Inhibition, and cAMP-Dependent Vasodilator-Stimulated Phosphoprotein Stimulation. Evidence-Based Complementary and Alternative Medicine.

[22] George S, Brat P, Alter P, Amiot MJ (2005). Rapid determination of polyphenols and vitamin C in plant-derived products. J Agric Food Chem.

[23] OECD (2015). OECD guidelines for testing of chemicals, test no. 439: *in vitro* skin irritation: reconstructed human epidermis test method.

[24] OECD, 2017. OECD guidelines for testing of chemicals, test no. 437: bovine corneal opacity and permeability test method for identifying i) chemicals inducing serious eye damage and ii) chemicals not requiring classification for eye irritation or serious eye damage.

[25] NIH/ICCVAM (2006). ICCVAM test methods evaluation report: *in vitro* ocular toxicity test methods for identifying severe irritants and corrosives.

[26] Pande G, Akoh CC (2009). Antioxidant capacity and lipid characterization of six Georgia-grown pomegranate cultivars. J Agric Food Chem.

[27] Ahumada J, Fuentealba C, Olaeta JA, Undurraga P, Pedreschi R, Shetty K, Chirinos R, Campos D, Ranilla LG (2017). Bioactive compounds of loquat (*Eriobotrya japonica* Lindl.) cv. Golden nugget and analysis of *in vitro* functionality for hyperglycemia management. Cien Inv Agr.

[28] Kim SJ, Park JN, Park SN (2012). Antioxidative effect and component analysis of *Eriobotrya japonica* leaf extracts. J Soc Cosmet Sci Korea.

[29] Park YH, Kim JH, Choi JH, Park SY (2009). Effects of *Eriobotryae folium* as anti-oxidant on HaCaT keratinocyte. J Korean Med Ophthalmol Otolaryngol Dermatol.

[30] Local Food-Nutraceuticals Consortium (2005). Understanding local Mediterranean diets: a multidisciplinary pharmacological and ethnobotanical approach. Pharmacol Res.

[31] Trouba KJ, Hamadeh HK, Amin RP, Germolec DR (2002). Oxidative stress and its role in skin disease. Antioxid Redox Signal.

[32] Braca A, Sortino C, Politi M, Morelli I, Mendez J (2002). Antioxidant activity of flavonoids from Licania licaniaeflora. J Ethnopharmacol.

[33] Badami S, Gupta MK, Suresh B (2003). Antioxidant activity of the ethanolic extract of Striga orobanchioides. J Ethnopharmacol.

[34] Korhonen R, Lahti A, Kankaanranta H, Moilanen E (2005). Nitric oxide production and signaling in inflammation. Curr Drug Targets Inflamm Allergy.

[35] Bradley JR (2008). TNF-mediated inflammatory disease. J Pathol.

[36] Cha DS, Eun JS, Jeon H (2011). Anti-inflammatory and antinociceptive properties of the leaves of *Eriobotrya japonica*. J Ethnopharmacol.

[37] Liu Y, Zhang W, Xu C, Li X (2016). Biological activities of extracts from loquat (*Eriobotrya japonica* Lindl.): a review. Int J Mol Sci.

[38] Agar N, Young AR (2005). Melanogenesis: a photoprotective response to DNA damage?. Mutat Res.

[39] Betteridge DJ (2000). What is oxidative stress?. Metabolism.

[40] Urabe K, Nakayama J, Hori Y, Norlund JJ, Boissy RE, Hearing VJ (1998). Mixed epidermal and dermal hypermelanoses. The pigmentary system: physiology and pathophysiology.

[41] Cullen MK, Norlund JJ, Biossy RE, Hearing VJ (1998). Genetic epidermal syndromes: disorders characterized by lentigines. The pigmentary system: physiology and pathophysiology.

[42] Tan H, Sonam T, Shimizu K (2017). The potential of triterpenoids from loquat leaves (*Eribotrya japonica*) for prevention and treatment of skin disorder. Int J Mol Sci.

[43] Park JO, Park JO, Joo CG (2015). A study on whitening and anti-inflammatory effects of *Eriobotrya japonica* leaf extracts with different extraction methods. J Soc Cosmet Sci Korea.

[44] Christian L, Freia FS, Angela R, Szymon K, Daniel K, Bart DW, Heike W, Florian KG (2016). Alternative methods for the replacement of eye irritation testing. ALTEX.

